# Parathyroid Carcinoma Presenting as Recurrent Primary Hyperparathyroidism and Neck Mass: A Case Report

**DOI:** 10.17925/EE.2023.19.2.6

**Published:** 2023-07-11

**Authors:** Hiya Boro, Harish Sharma, Deepak Mittal, Mohit Pareek, Shilpa Chugh, Mohar Singh Jakhar, Neeraj Nagar, Lovekesh Bhatia, Sanjay Saini, Vashishth Joshi, Sahil Vaid, Velmurugan Mannar, Lakshmi Nagendra, Mazhar Dalvi, Vikash Bundela

**Affiliations:** 1. Department of Endocrinology and Metabolism, Aadhar Health Institute, Hisar, Haryana, India; 2. Department of Surgery, Aadhar Health Institute, Hisar, Haryana, India; 3. Department of Otorhinolaryngology, Aadhar Health Institute, Hisar, Haryana, India; 4. Department of Pathology, Aadhar Health Institute, Hisar, Haryana, India; 5. Department of Anaesthesiology and Critical Care, Aadhar Health Institute, Hisar, Haryana, India; 6. Department of Radiodiagnosis, Aadhar Health Institute, Hisar, Haryana, India; 7. Department of Endocrinology, Aster Clinic, Dubai, United Arab Emirates; 8. Department of Endocrinology, Jagadguru Sri Shivarathreeshwara Medical College, Mysuru, India; 9. Department of Endocrinology, Al Noor Mediclinic, Abu Dhabi, United Arab Emirates; 10. Department of Gastroenterology, Aadhar Health Institute, Hisar, Haryana, India

**Keywords:** Case report, endocrine neoplasm, hypercalcaemia, neck dissection, neck mass, parathyroid carcinoma, primary hyperparathyroidism, radiotherapy

## Abstract

Parathyroid carcinoma is a rare endocrine neoplasm that accounts for <1% of cases of primary hyperparathyroidism. The management of parathyroid carcinoma is a challenge due to the high rate of local recurrence of the tumour. We report the case of a middle-aged north Indian woman who presented with recurrent primary hyperparathyroidism due to parathyroid carcinoma. She presented with a recurrent palpable hard neck mass and underwent radical dissection of the neck six times. At the time of writing this report, she was referred for external beam radiotherapy to the neck. Parathyroid carcinoma is a rare malignancy with an indolent but tenacious course. Complete resection at the time of initial surgery determines the prognosis of the neoplasm. Chemotherapy and radiotherapy are usually ineffective. Hypercalcaemia needs to be aggressively managed. A multidisciplinary team is required to effectively manage parathyroid carcinoma.

Parathyroid carcinoma is a rare endocrine neoplasm with an incidence of 0.5–2.0% of all cases of primary hyperparathyroidism (PHPT).^1^ It was first described in 1904 by de Quevain, when it was found in a patient presenting with a non-functioning parathyroid mass.^2^ Around 26 years later, Sainton and Millot described the first functioning parathyroid carcinoma.^3^

There is no known predisposing factor for parathyroid carcinoma. Patients usually present in their 50s, which is a decade earlier than is usual for benign parathyroid adenomas.^1^ Men and women are equally affected by parathyroid carcinoma, unlike in benign PHPT, which is 3–4 times more common in women.^1^ Parathyroid carcinoma may occur sporadically or may be a result of a genetic syndrome such as autosomal dominant isolated familial hyperparathyroidism.^4^ It can also be seen in 15% of cases of hyperparathyroidism-j aw tumour syndrome due to mutation in the hyperparathyroidism 2 (*HRPT2*) gene encoding the protein parafibromin.^5^ In addition, other chromosomal abnormalities identified are reciprocal translocation between chromosomes 3 and 4, trisomy 7, loss of chromosome 13q, pericentric inversion of chromosome 9, and somatic mutations of genes such as cyclin D1 (*CCND1*), retinoblastoma (*RB*) and tumour protein P53 (*TP53*).^5^ The genetic syndromes of multiple endocrine neoplasia type 1 (MEN1) and type 2A (MEN2A) are usually not associated with parathyroid carcinoma.^5^

Parathyroid carcinoma may mimic benign cases of PHPT and cause diagnostic challenges.^6^ Preoperatively, it may be difficult to distinguish between benign and malignant cases based on history and imaging. Even in histopathology, it may be difficult to distinguish parathyroid carcinoma from atypical parathyroid adenoma. Managing parathyroid carcinoma is also difficult, since there is a high recurrence rate with no definitive role for radiotherapy or chemotherapy.

In this case report, we describe a patient with a history of recurrent PHPT due to parathyroid carcinoma. We aim to increase awareness of the challenges encountered in parathyroid carcinoma and to highlight the importance of a thorough and individualized approach. This report also contributes to the limited medical literature on parathyroid carcinoma and provides valuable insights for healthcare professionals, who may encounter similar cases in their practice.

**Figure 1:**
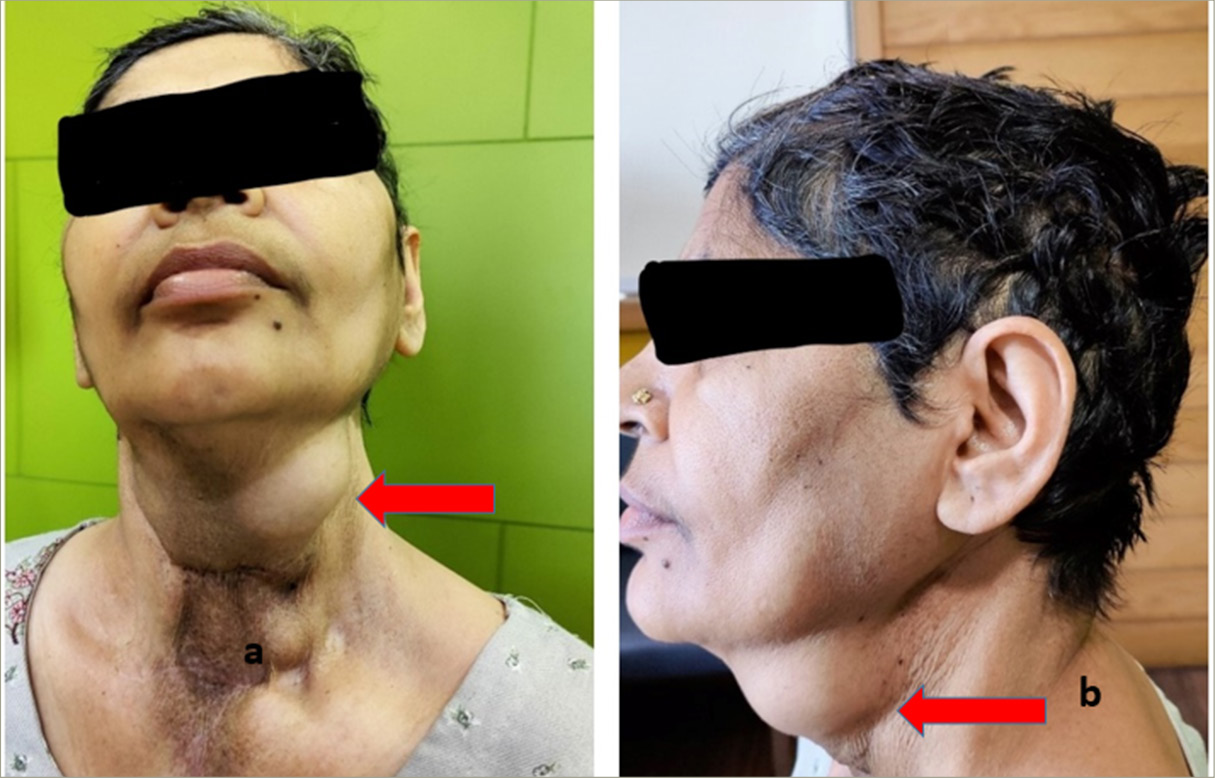
Clinical images of the patient showing a neck mass (arrows) with previous surgical scars from the front (a) and left side (b)

## Case report

A 55-year-old woman from north India presented to our endocrine clinic with a history of recurrent neck mass for the past 6 years. It started as a small hard nodule in the front of her neck that rapidly progressed in size. There was no pain, redness, fever, difficulty in swallowing, difficulty in breathing, stridor or hoarseness of voice. The mass did not move with deglutition or with the movement of the tongue. Laboratory investigations suggested PHPT with elevated serum calcium of 3.62 mmol/L (normal range [N]: 2.12–2.62 mmol/L) and elevated serum intact parathyroid hormone (iPTH) of 142.21 pmol/L (N: 1.59–6.89 pmol/L). She underwent the first surgical resection of the mass 6 years ago, and the histopathology confirmed a diagnosis of parathyroid carcinoma.

Around a year after the first surgery, the mass recurred in the same region of the neck. It again progressed rapidly in size and was hard in consistency. Biochemically, she again had PHPT. She underwent a second surgery, which was followed by a relapse 6 months later. In this manner, over the course of 6 years, she underwent five neck surgeries, which involved radical neck dissection with the removal of the entire mass, the thyroid with both its lobes and isthmus, and all the parathyroid glands along with lymph node dissection.

The current presentation was the recurrence of the neck mass for the sixth time (*Figure 1a and b*). On examination, it was a large mass of 5 x 4 cm that involved the anterior aspect of the mid and left side of the neck and extended inferiorly into the anterior part of the sternum. It was hard in consistency and did not move with deglutition or protrusion of the tongue. It was non-tender and there was no redness or ulceration of the skin over the mass. Scars from previous surgeries were evident and were clean. Systemic examination was normal with no localized bone tenderness. Her height was 172.7 cm and weight was 85.0 kg with a body mass index of 28.8 kg/m^2^.

In this presentation, the patient also reported a loss of appetite for 2 months. She had multiple episodes of vomiting during the 2 weeks prior to admission, along with vague abdominal pain and constipation. For the past 6 years, she experienced diffuse musculoskeletal pain and low backache. There was no history of weight loss, fracture, bone deformity or proximal myopathy. She had a history of recurrent renal stones and had undergone percutaneous nephrolithotomy three times previously. She had depressive symptoms including low mood, sadness and crying spells. There was no history suggestive of any syndromic disorder like galactorrhoea, enlargement of hands and feet (MEN1), adrenergic spells (MEN2A), jaw tumour (hyperparathyroidism-j aw tumour syndrome), or any family history of PHPT (isolated familial hyperparathyroidism).

**Table 1:** Biochemical parameters of the patient before and after surgery

Parameter	Normal range	Pre-operative	Post-operative after 1 week
Serum calcium, mmol/L	2.12–2.62	3.74	1.97
Serum phosphate, mmol/L	0.81–1.45	0.67	0.83
Serum alkaline phosphatase, IU/L	80–240	456	589
Total protein, g/L	64–83	78	N/A
Serum albumin, g/L	35–52	43	N/A
Serum creatinine, μmol/L	44.21–97.26	70.72	N/A
Intact parathyroid hormone, pmol/L	1.59–6.89	88.65	8.64
25-hydroxy-vitamin D, nmol/L	50.0–125.0	32.7	N/A
Thyroxine, nmol/L	64.5–160.6	104.2	N/A
Thyroid-stimulating hormone, mIU/L	0.55–4.78	2.18	1.21
24 h urinary calcium, mmol/24 h	2.5–7.5	15.0	N/A

*N/A = Not applicable.*

Biochemistry revealed serum calcium of 3.74 mmol/L, serum inorganic phosphate (PO_4_) of 0.67 mmol/L (N: 0.81–1.45 mmol/L), serum alkaline phosphatase of 456 IU/L (N: 80–240 IU/L), serum total protein of 78 g/L (N: 64–83 g/L), serum albumin of 43 g/L (N: 35–52 g/L), serum creatinine of 70.72 μmol/L (N: 44.21–97.26 μmol/L), serum iPTH of 88.65 pmol/L, and serum 25-hydroxy-vitamin D of 32.7 nmol/L (N: 50.0–125.0 nmol/L) (*Table 1*). Urine routine examination was normal, while 24 h urinary calcium was 15.0 mmol/24 h (N: 2.5–7.5 mmol/24 h). Thyroid function tests were normal: thyroxine was 104.2 nmol/L (N: 64.5–160.6 nmol/L) and serum thyroid-stimulating hormone was 2.18 mIU/L (N: 0.55–4.78 mIU/L). She was taking thyroxine at a dose of 125 μg daily. Her complete blood count and liver function tests were also within normal limits (haemoglobin 7.51 mmol/L [N: 7.45–9.31 mmol/L]; total bilirubin 8.31 μmol/L [N: 5.13–17.10 μmol/L]; alanine transaminase 32 U/L [N: 4–36 U/L]). Her fasting plasma glucose was also normal at 4.1 mmol/L (N: 3.1–5.6 mmol/L).

Ultrasonography of the kidneys revealed bilateral nephrocalcinosis. A skeletal survey revealed subperiosteal resorption of phalanges and intracortical tunnelling, although there was no salt and pepper appearance of the skull, osteitis fibrosa cystica or bone fracture/pseudo-fracture. Bone mineral density (BMD) measured by dual-energy X-ray absorptiometry (Hologic, Marlborough, MA, USA) revealed osteopenia at the lumbar spine (L_1_–L_4_; BMD T score -2.3), femoral neck (BMD T score -2.1) and total hip (BMD T score -1.6), and osteoporosis at the distal forearm/radius (T score -4.2).

**Figure 2:**
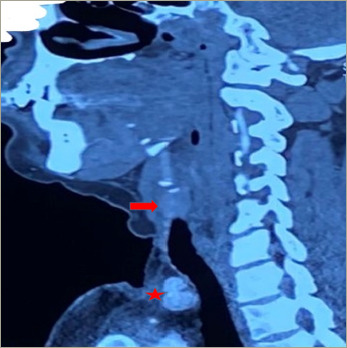
Contrast-enhanced computed tomography images (sagittal view) showing an oval-shaped heterogeneously enhancing lesion on the left side of the neck (arrow). Two adjacent heterogeneously enhancing lymph nodes are seen in the left supraclavicular region (star)

Ultrasonography of the neck revealed post-total thyroidectomy status. It showed two heterogeneously placed lesions on the left side of the neck, approximately 4.5 x 3.9 cm and 2.9 x 2.2 cm. Another lesion of the same morphology was also seen in the left supraclavicular/lower cervical region, which was reported as a lymph node. Cytology was not done due to the risk of seeding of the tumour through the needle track. Contrast-enhanced computed tomography of the neck revealed a well defined round-to-oval lesion with lobulated margins in the visceral space of the infrahyoid part of the neck (*Figure 2*). The lesion measured approximately 2.0 x 2.1 x 2.6 cm and showed heterogenous post-contrast enhancement with central non-enhancing necrotic areas. Two heterogenously enhancing lymph nodes were seen in the left supraclavicular level, the largest measuring 1.3 x 1.5 cm. Technetium (^99m^Tc) sestamibi parathyroid imaging showed sestamibi avid focal areas in the left side of the neck and left supraclavicular region.

**Figure 3:**
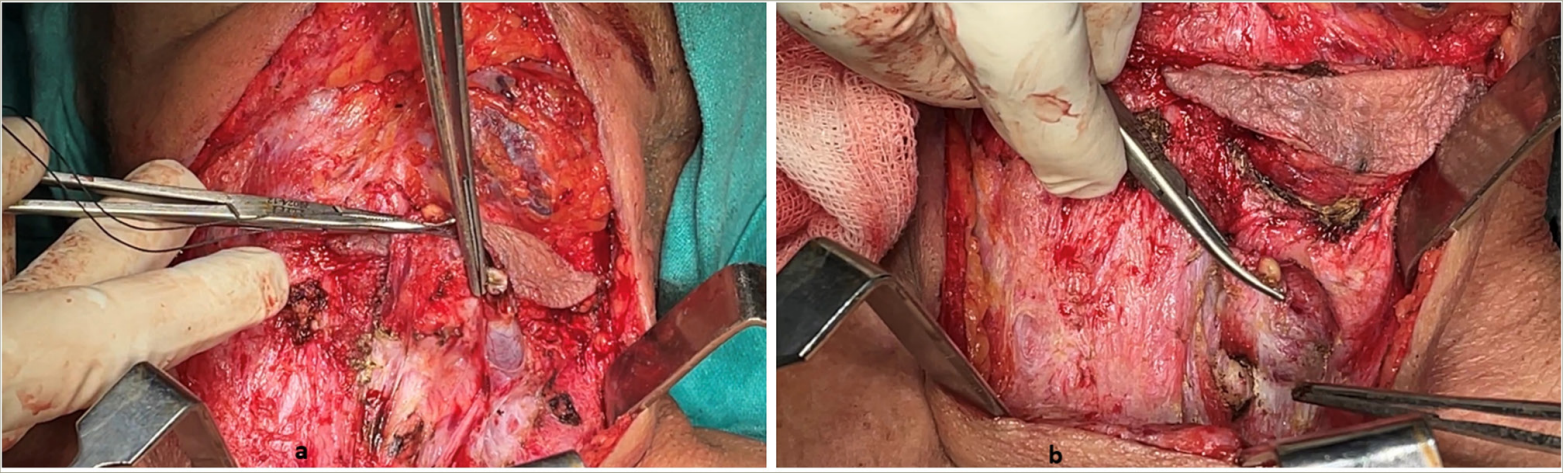
Intra-operative images showing a large neck mass adhering to adjacent structures

**Figure 4:**
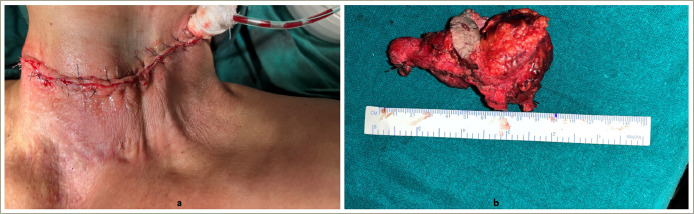
Post-operative images showing the scar on the neck of the patient (a) and the gross tumour specimen (b)

She was admitted to our hospital and hypercalcaemia was managed immediately. Saline diuresis with 100 mL/h of isotonic 0.9% normal saline and 160 mg of intravenous furosemide was started, alongside 200 IU of subcutaneous calcitonin every 6 hours and a single dose of 4 mg intravenous zoledronic acid. After all these measures, serum calcium dropped to 3.37 mmol/L, and the patient was taken for surgery (2 days after admission). The neck mass was dissected on the left side. The mass was adhering to the left internal jugular vein and the inferior constrictor of the pharynx, which were removed along with the mass. The retroclavicular portion of the mass was also carefully excised. The intra-operative and immediate post-operative images are shown in *Figures 3 and 4*.

**Figure 5:**
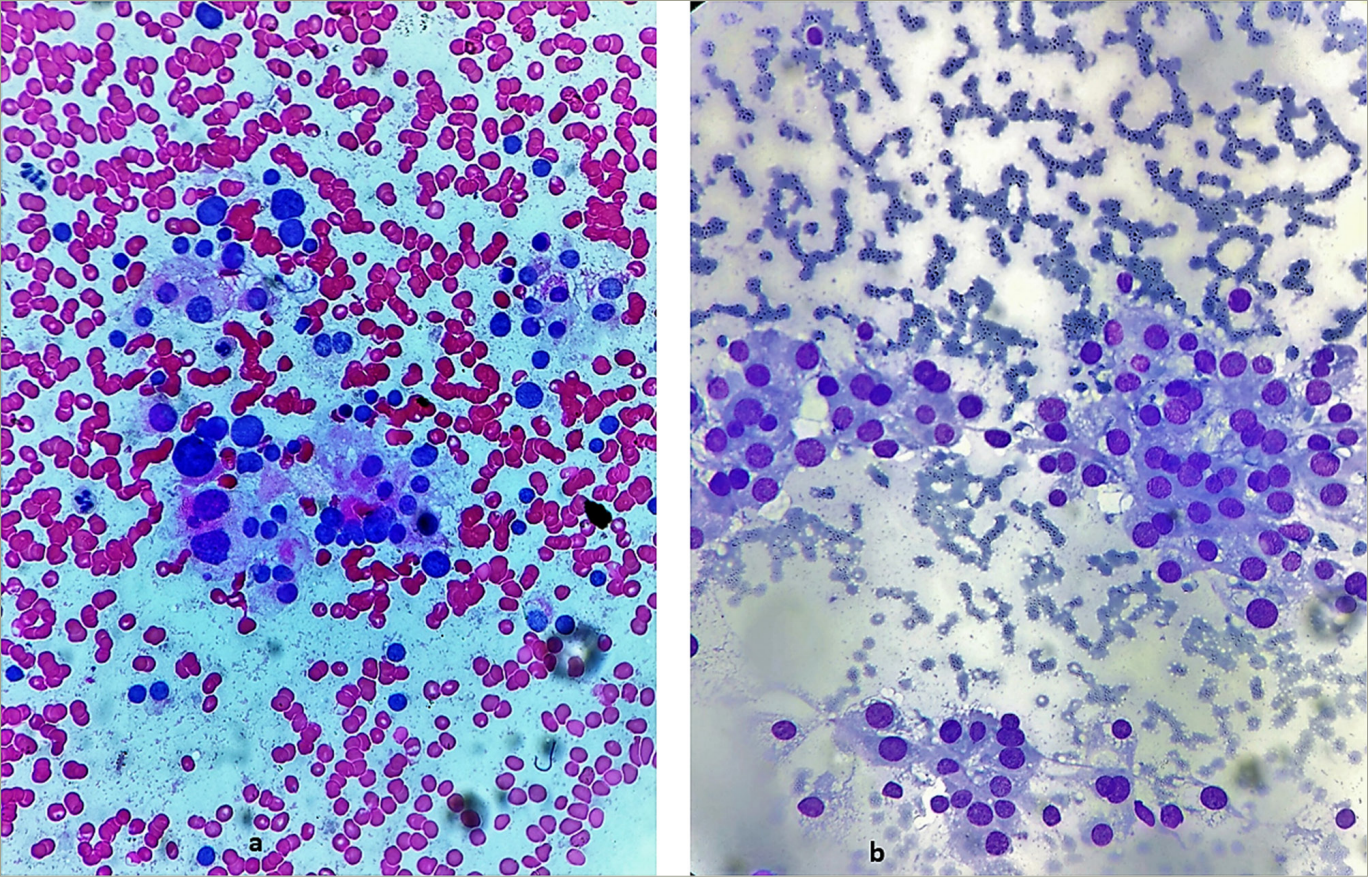
Histopathology images showing tumour cells separated by broad fibrotic bands (a). The tumour cells were uniform with mild to moderate nuclear atypia and macro-nucleoli with 10–15 mitosis/10 high power field. Atypical mitotic figures and capsular and vascular invasion were also observed (b)

Post-surgery, serum calcium dropped to 2.87 mmol/L and serum iPTH to 24.49 pmol/L. The patient was discharged in stable condition and underwent repeat serum calcium testing and repeat serum iPTH testing after a week. Histopathology suggested nodular growth patterns separated by broad fibrotic bands (*Figure 5a and b*). The tumour cells were uniform with mild to moderate nuclear atypia and macro-nucleoli with 10–15 mitosis/10 high power field. Atypical mitotic figures and capsular and vascular invasion were also observed. There was, however, no perineural invasion.

One week after surgery, the patient's serum calcium was 1.97 mmol/L and iPTH was 8.64 pmol/L (*Table 1*). She was started on calcium supplements with 1 g of elemental calcium and 60,000 IU of cholecalciferol once a week for 8 weeks. At the time of writing this report (four weeks after surgery), after discussion with a multidisciplinary team, the patient was planned for external-beam radiotherapy to prevent local regrowth of the tumour.

## Discussion

The current case represents a unique clinical presentation of recurrent PHPT due to parathyroid carcinoma. A palpable neck mass has been reported in 30–76% of patients with parathyroid carcinoma.^1^ Clinically, there is no definitive symptom or sign that can differentiate between benign PHPT and parathyroid carcinoma. Usually, patients with parathyroid carcinoma tend to have more severe skeletal and renal symptoms due to a more severe degree of hypercalcaemia.^7^

A case series has reported a prevalence of nephrolithiasis in 56% and renal insufficiency in 84% of patients with parathyroid carcinoma.^8^ The classical radiological manifestations of skeletal involvement like osteitis fibrosa cystica, salt and pepper appearance of the skull, subperiosteal resorption of phalanges, and loss of lamina dura of the teeth are more commonly seen in parathyroid carcinoma, although they may be present in severe benign PHPT, particularly in developing countries.^7^ In our patient, renal involvement was predominant with recurrent renal stones, while florid skeletal disease was absent.

Pathologically, parathyroid carcinoma poses a diagnostic challenge. The World Health Organization 2022 classification of parathyroid tumours requires one of the following findings to be present for histological diagnosis of parathyroid carcinoma: (i) angio-invasion, characterized by a tumour invading the vessel wall, and associated thrombus or intravascular tumour cells admixed with thrombus; (ii) lymphatic invasion; (iii) perineural invasion; (iv) local malignant invasion into adjacent structures; or (v) histologically/cytologically documented metastatic disease.^9^ It is recommended to measure mitotic activity per high power field and Ki-67 labelling index in parathyroid carcinomas.^9^ In our patient, the mass invaded adjacent structures, although there was no distant metastasis. The tumour specimen also demonstrated a high mitotic index, suggestive of its malignant potential.

The management of parathyroid carcinoma is an arduous task.^10^ Complete surgical resection with tumour-free margins offers the best possible cure. Certain intra-operative findings may arouse suspicion of parathyroid carcinoma, such as large size, greyish to whitish colour and adherence to adjacent structures like the strap muscles, thyroid gland, recurrent laryngeal nerve, trachea or oesophagus.^10^ Upon recognizing malignant features during surgery, the surgeon should perform en bloc resection of all the tissues involved by the tumour, taking great care not to breach the tumour capsule.^10^ It has been recommended to remove the adjacent thyroid lobe; however, previous studies show that this does not improve survival in parathyroid carcinoma.^11,12^

In the immediate post-operative period, if the histopathology suggests parathyroid carcinoma and the patient remains hypercalcaemic, re-exploration of the neck may be required.^10^ If the patient attains normal serum calcium levels in the post-operative period and parathyroid carcinoma is diagnosed based on microscopic features, immediate re-exploration may not be required, rather it becomes imperative to frequently monitor serum calcium.^10^ In the current case, we do not have records of post-operative calcium after the initial surgery. The patient's history shows that she had persistent disease and could have benefitted from immediate re-exploration after histopathology confirmed the diagnosis.

Hungry bone syndrome is more commonly seen in parathyroid carcinoma, due to more severe skeletal disease and profound hypercalcaemia. In the immediate post-operative period, patients may require intravenous calcium infusion and calcitriol. As the other parathyroid glands recover, the requirement of calcium and calcitriol reduces. Our patient did not have hungry bone syndrome, which might be attributed to less severe skeletal presentation prior to surgery.

The management of recurrent or metastatic parathyroid carcinoma is primarily surgical. Unlike other cancers, it has an indolent nature and is mostly associated with local recurrence and involvement of contiguous structures in the neck rather than distant metastasis. The latter, if present, is mostly observed in the lymph nodes, lungs or liver.^6^ Tumour deposits at any site, even though small, can produce sufficient parathyroid hormone (PTH) to cause significant hypercalcaemia. In most cases, surgical removal of the metastatic deposits can ameliorate hypercalcaemia considerably, providing a window of opportunity for medical therapies to control remaining hypercalcaemia.^10^

In patients with recurrence of parathyroid carcinoma, careful localization should be done with ^99m^Tc sestamibi scan, computed tomography or magnetic resonance imaging of the neck and chest. Biopsy or fine needle aspiration cytology should not be done to avoid seeding of the tumour.^10^ Recurrent parathyroid carcinoma in the neck should be managed with wide local excision, removing regional lymph nodes and all the adjacent involved structures. Distant metastatic deposits, if accessible, should be resected.^10^

Radiotherapy in parathyroid carcinoma is ineffective in the majority of cases, as it is not a radio-sensitive tumour.^10^ A few case series have, however, found that radiotherapy to the neck post-surgery prevented tumour regrowth in the local areas.^7,13^ Wynne et al. reported a tumour-free state for 10 years following adjuvant radiotherapy after surgery for parathyroid carcinoma.^14^ Usually, the decision for radiotherapy should be made on an individual basis. In our patient, radical neck dissection had been done six times. However, re-surgery is rarely curative and relapse is likely, as has been observed in our patient. Such patients usually have a 60% lifetime accumulated surgical risk from all subsequent surgical interventions.^11,15^ Therefore, in our multidisciplinary team meeting, it was decided to offer radiotherapy to the patient based on previous anecdotal reports.^7,13,14^

The role of chemotherapy in parathyroid carcinoma is a matter of debate. The results of chemotherapy in parathyroid carcinoma have been disappointing. Several chemotherapy regimens (vincristine, cyclophosphamide and actinomycin D; adriamycin, cyclophosphamide and 5-fluorouracil; and adriamycin alone) have been ineffective.^10,16–18^ Bukowski et al. reported a single patient with pulmonary metastases responding to a chemotherapy regimen of dacarbazine, 5-fl uorouracil and cyclophosphamide with normalization of serum calcium for 13 months.^16^

The management of hypercalcaemia in parathyroid carcinoma can be difficult due to the severity of hypercalcaemia. Management of hypercalcaemia includes saline infusion to replenish volume status followed by loop diuretics to increase urinary calcium excretion. Such measures are usually not sufficient and agents inhibiting osteoclastic bone resorption, such as bisphosphonates, denosumab and calcitonin, are usually required.^19^ Among bisphosphonates, the most potent agent, 4 mg zoledronic acid by intravenous infusion, is usually administered. It may cause transient side effects such as fever, chills, exacerbation of bone pain, and very rarely, cardiac arrhythmias.^20^ Denosumab, a monoclonal antibody against receptor activator of nuclear factor κΒ ligand (RANKL), inhibits osteoclastic bone resorption and is used to manage hypercalcaemia in parathyroid carcinoma.^21,22^ Calcitonin inhibits osteoclast-mediated bone resorption and increases urinary calcium. However, the reduction in calcium is transient due to the development of tachyphylaxis.^23^ Cinacalcet is a calcimimetic agent that is an allosteric modulator of the calcium-sensing receptor protein, which lowers serum PTH and calcium levels in PHPT. Cinacalcet has been tried in cases of parathyroid carcinoma, with a transient reduction in serum calcium level.^24,25^ One case report has described the efficacy of octreotide, a long-acting somatostatin analogue, in a woman with parathyroid carcinoma and metastatic bone disease, where they show a transient decrease in PTH levels.^26^ Synthetic oestrogen has been found to be successful in a single case.^27^ Immunization with human and bovine PTH peptides with booster doses at 4 and 11 weeks helped lower serum calcium levels for 6 months in a patient with parathyroid carcinoma.^28,29^

Parathyroid carcinoma has a variable prognosis. Hypercalcaemia seems to be the primary cause of mortality and morbidity in these patients.^30^ The single most important factor affecting prognosis is the extent of resection at the time of initial surgery.^31^ Usually, a gap of 3 years is noted between the initial surgery and the first recurrence, although it can occur even after 20 years.^1^ Once there is a recurrence, palliative surgery can be of considerable benefit, although a complete cure is unlikely.

## Conclusion

We have described a case of recurrent PHPT due to parathyroid carcinoma in a woman with a recurrent neck mass. She underwent palliative surgery multiple times and, at the time of writing this report, was referred for external radiotherapy. Parathyroid carcinoma imposes a considerable challenge in terms of diagnosis and management. Parathyroid carcinoma should be considered in patients presenting with severe symptomatic disease, neck mass, and significantly elevated calcium and PTH levels. A multidisciplinary team is required to effectively manage parathyroid carcinoma. This case report also emphasizes the lack of options in treating these types of cancers, and there is a definitive need for future research in targeted therapies.

## References

[1] Shane E (2001). Clinical review 122: Parathyroid carcinoma.. J Clin Endocrinol Metab..

[2] De Quevain F (1904). [Malignant aberrant parathyroid].. Dtsch Z Fuer Chir..

[3] Sainton P, Millot J (1933). [Malignant eosinophilic parathyroid].. Au cours d’une de Recklinghausen Annales Anatomie Pathologique..

[4] Yoshimoto K, Endo H, Tsuyuguchi M (1998). Familial isolated primary hyperparathyroidism with parathyroid carcinomas: Clinical and molecular features.. Clin Endocrinol (Oxf)..

[5] Thakker RV (2016). Genetics of parathyroid tumours.. J Intern Med..

[6] Marcocci C, Cetani F, Rubin MR (2008). Parathyroid carcinoma.. J Bone Miner Res..

[7] Levin KE, Galante M, Clark OH (1987). Parathyroid carcinoma versus parathyroid adenoma in patients with profound hypercalcemia.. Surgery..

[8] Silverberg SJ, Shane E, Jacobs TP (1990). Nephrolithiasis and bone involvement in primary hyperparathyroidism.. Am J Med..

[9] Erickson LA, Mete O, Juhlin CC (2022). Overview of the 2022 WHO classification of parathyroid tumors.. Endocr Pathol..

[10] Wei CH, Harari A (2012). Parathyroid carcinoma: Update and guidelines for management.. Curr Treat Options Oncol..

[11] Harari A, Waring A, Fernandez-Ranvier G (2011). Parathyroid carcinoma: A 43-year outcome and survival analysis.. J Clin Endocrinol Metab..

[12] Busaidy NL, Jimenez C, Habra MA (2004). Parathyroid carcinoma: A 22-year experience.. Head Neck..

[13] Chow E, Tsang RW, Brierley JD, Filice S (1998). Parathyroid carcinoma--the Princess Margaret Hospital experience.. Int J Radiat Oncol Biol Phys..

[14] Wynne AG, van Heerden J, Carney JA, Fitzpatrick LA (1992). Parathyroid carcinoma: Clinical and pathologic features in 43 patients.. Medicine (Baltimore)..

[15] Kebebew E, Arici C, Duh QY, Clark OH (2001). Localization and reoperation results for persistent and recurrent parathyroid carcinoma.. Arch Surg..

[16] Bukowski RM, Sheeler L, Cunningham J, Esselstyn C (1984). Successful combination chemotherapy for metastatic parathyroid carcinoma.. Arch Intern Med..

[17] Calandra D, Shah K, Lawrence AM, Paloyan E (1985). Parathyroid carcinoma. A report of five cases.. Am Surg..

[18] Alberti A, Smussi D, Zamparini M (2022). Treatment and outcome of metastatic parathyroid carcinoma: A systematic review and pooled analysis of published cases.. Front Oncol..

[19] Seisa MO, Nayfeh T, Hasan B (2023). A systematic review supporting the Endocrine Society Clinical Practice Guideline on the treatment of hypercalcemia of malignancy in adults.. J Clin Endocrinol Metab..

[20] Reid IR, Green JR, Lyles KW (2020). Zoledronate.. Bone..

[21] Roukain A, Alwan H, Bongiovanni M (2021). Denosumab for the treatment of hypercalcemia in a patient with parathyroid carcinoma: A case report.. Front Endocrinol (Lausanne)..

[22] Fountas A, Andrikoula M, Giotaki Z (2015). The emerging role of denosumab in the long-term management of parathyroid carcinoma-related refractory hypercalcemia.. Endocr Pract..

[23] Mirrakhimov AE (2015). Hypercalcemia of malignancy: An update on pathogenesis and management.. N Am J Med Sci..

[24] Nadarasa K, Bailey M, Chahal H (2014). The use of cinacalcet in pregnancy to treat a complex case of parathyroid carcinoma.. Endocrinol Diabetes Metab Case Rep..

[25] Silverberg SJ, Rubin MR, Faiman C (2007). Cinacalcet hydrochloride reduces the serum calcium concentration in inoperable parathyroid carcinoma.. J Clin Endocrinol Metab..

[26] Koyano H, Shishiba Y, Shimizu T (1994). Successful treatment by surgical removal of bone metastasis producing PTH: New approach to the management of metastatic parathyroid carcinoma.. Intern Med..

[27] Sigurdsson G, Woodhouse NJ, Taylor S, Joplin GF (1973). Stilboestrol diphosphate in hypercalcaemia due to parathyroid carcinoma.. Br Med J..

[28] Sarquis M, Marx SJ, Beckers A (2020). Long-term remission of disseminated parathyroid cancer following immunotherapy.. Endocrine..

[29] Betea D, Bradwell AR, Harvey TC (2004). Hormonal and biochemical normalization and tumor shrinkage induced by anti-parathyroid hormone immunotherapy in a patient with metastatic parathyroid carcinoma.. J Clin Endocrinol Metab..

[30] Shahar S, Lim KP, Mohamad M (2019). Parathyroid carcinoma: Analysis of patient characteristics and outcomes in a retrospective review of eight cases seen in a single center.. J ASEAN Fed Endocr Soc..

[31] Ullah A, Khan J, Waheed A (2022). Parathyroid carcinoma: Incidence, survival analysis, and management: A study from the SEER database and insights into future therapeutic perspectives.. Cancers (Basel)..

