# Erratum to Correct Informed Patient Consent Statement

**DOI:** 10.17925/EE.2024.20.1.2

**Published:** 2023-12-07

**Authors:** Hiya Boro, Harish Sharma, Deepak Mittal, Mohit Pareek, Shilpa Chugh, Mohar Singh Jakhar, Neeraj Nagar, Lovekesh Bhatia, Sanjay Saini, Vashishth Joshi, Sahil Vaid, Velmurugan Mannar, Lakshmi Nagendra, Mazhar Dalv, Vikash Bundela

**Affiliations:** 1. Department of Endocrinology and Metabolism, Aadhar Health Institute, Hisar, Haryana, India; 2. Department of Surgery, Aadhar Health Institute, Hisar, Haryana, India; 3. Department of Otorhinolaryngology, Aadhar Health Institute, Hisar, Haryana, India; 4. Department of Pathology, Aadhar Health Institute, Hisar, Haryana, India; 5. Department of Anaesthesiology and Critical Care, Aadhar Health Institute, Hisar, Haryana, India; 6. Department of Radiodiagnosis, Aadhar Health Institute, Hisar, Haryana, India; 7. Department of Endocrinology, Aster Clinic, Dubai, United Arab Emirates; 8. Department of Endocrinology, Jagadguru Sri Shivarathreeshwara Medical College, Mysuru, India; 9. Department of Endocrinology, Al Noor Mediclinic, Abu Dhabi, United Arab Emirates; 10. Department of Gastroenterology, Aadhar Health Institute, Hisar, Haryana, India

This corrects the article: “Boro H, Sharma H, Mittal D, et al. Parathyroid Carcinoma Presenting as Recurrent Primary Hyperparathyroidism and Neck Mass: A Case Report. touchREVIEWS in Endocrinology. 2023;19(2):80–85”

The patient consent statement was added incorrectly due to an editorial error. The correct patient informed consent statement, provided by the authors, is included below.

“This study was performed in accordance with the Helsinki Declaration of 1964, and its later amendments. Ethics approval is not required as case reports are not deemed to constitute research by the Aadhar Health Institute Review Board. Written informed consent was obtained from the patient for publication of the manuscript along with the images.”

